# Efficacy and Safety of Endoscopic Submucosal Dissection for Dysplasia in Ulcerative Colitis Patients: A Systematic Review and Meta-Analysis

**DOI:** 10.1155/2022/9556161

**Published:** 2022-01-25

**Authors:** Qi-Shan Zeng, Zhi-Jing Zhao, Jiao Nie, Min Zou, Jia-Hui Yang, Jin-Zhi Zhang, Hua-Tian Gan

**Affiliations:** ^1^Department of Gastroenterology and the Center of Inflammatory Bowel Disease, West China Hospital, Sichuan University, Chengdu, Sichuan Province, China; ^2^Department of Gastroenterology, The Sixth People's Hospital of Chengdu, Sichuan Province, China; ^3^Department of Gastroenterology and National Clinical Research Center for Geriatrics, West China Hospital, Sichuan University, Chengdu, Sichuan Province, China

## Abstract

**Background and Aims:**

Ulcerative colitis (UC) is associated with an increased risk of colorectal cancer. Current guidelines recommend endoscopic resection if the lesion is visible with distinct margins and a complete resection can be achieved. However, submucosal fibrosis due to chronic inflammation may increase the procedural risk and reduce the complete resection rate. The aim of this study is to assess the efficacy and safety of endoscopic submucosal dissection (ESD) for dysplasia in UC patients.

**Materials and Methods:**

A systematic search of databases was performed until May 30, 2021. Studies that reported the resection rates and complication rates of ESD for dysplasia in UC patients were included. A random-effects model was used to generate conservative estimates of the prevalence of the outcome variables. All data analyses were performed using software Stata (version 15).

**Results:**

8 studies were enrolled in the meta-analysis, with a total of 203 dysplastic lesions in 192 UC patients. The mean lesion size was 26.7 mm. About 83% of the lesions were located in the left-side colon, and 90% of the lesions were nonpolypoid, and about 71% of the lesions had submucosal fibrosis. The mean procedural time of ESD was 83 minutes. The en bloc resection rate, complete resection rate, and curative resection rate were 94%, 84%, and 81%, respectively, with a local recurrence rate of 5%. The pooled prevalence of bleeding and perforation were 8% and 6%, respectively. The rates of metachronous tumors and additional surgery after ESD were 6% and 10%, respectively.

**Conclusion:**

Despite some limitations, our study suggests that ESD is an effective and safe treatment for dysplasia in UC patients. However, randomized controlled multicenter studies with less heterogeneity and longer follow-up are needed to better assess the clinical outcomes of ESD in UC patients.

## 1. Introduction

Ulcerative colitis (UC) is an idiopathic, long-lasting, and relapsing inflammatory bowel disease that is increasing in incidence in both Western countries and Asian areas [1]. Patients with ulcerative colitis carry a higher risk of developing colorectal cancer (CRC), varying with the duration and extent of the disease [2, 3]. Indeed, chronic inflammation of the colonic mucosa predisposes to the onset of dysplasia [4], which is a precursor of cancer. Therefore, endoscopic surveillance and treatment of dysplasia in UC patients, recommended by both ECCO and SCENIC guidelines [5, 6], is of great importance for the prevention of UC-related cancer.

Endoscopic submucosal dissection (ESD), since its first introduction in Japan 20 years ago, has become a safe and effective method to treat large, superficial neoplastic lesions [7]. Indeed, ESD allows en bloc resection regardless of lesion size and the severity of submucosal fibrosis [8], thus avoiding surgery in a definite proportion of patients [7]. Therefore, ESD might be considered an appropriate therapeutic option for dysplasia in UC patients. However, only a few case series have attempted to assess the outcomes of ESD for dysplasia in UC patients in recent years, so that the information is fragmentary, and a pooled data analysis would be useful. The aim of this study is to pool the results of ESD for dysplasia in UC patients to fully evaluate its efficacy and safety.

## 2. Materials and Methods

### 2.1. Search Strategy

This study was conducted following the meta-analysis of observational studies in epidemiology guidelines [9]. MEDLINE, Embase, Web of Science, Cochrane Library, and China National Knowledge Infrastructure (CNKI) were systematically searched for relevant studies published from inception until May 30, 2021. Search terms included MeSH term and keyword of “endoscopic submucosal dissection” and MeSH term and keyword of “ulcerative colitis.” Corresponding reference list of each included article was also reviewed in order to not to neglect any related study. In addition, the websites of Clinicaltrials.gov and Google Scholar were screened to make sure that gray literatures were evaluated.

### 2.2. Inclusion and Exclusion Criteria

Studies were included based on the following criteria: (1) patients diagnosed with UC and colorectal dysplasia; (2) ESD was performed for the dysplastic lesions in UC patients; (3) studies with/without control group; (4) clinical outcomes of ESD, such as resection rates and complications rates, were reported; (5) clinical trials including cohort, case-control, and randomized trials were enrolled. The following studies were excluded: (1) studies involved animal subjects; (2) hybrid ESD was performed; (3) studies that were case reports (less than 10 lesions), reviews, letters, editorials, or conference papers; and (4) full text not available. Two reviewers independently evaluated each study for eligibility, and any disagreements were resolved by discussion.

### 2.3. Data Extraction

Data extraction and quality assessment were also independently performed by two reviewers. Any disagreements were resolved by discussion. The following information were collected: (1) study and population characteristics, including name of the first author, publication time, country of origin, numbers of patients and lesions, distributions of age and gender, duration of UC, and study design and (2) technical and clinical characteristics, including en bloc resection rate, complete resection rate (R0 resection rate), curative resection rate, lesion size, location and morphology of lesions, extent and severity of colitis, submucosal fibrosis, procedural time, histopathological results, follow-up duration, prevalence of complications, and additional surgery after ESD.

The main outcome measures were en bloc resection, defined as complete removal of the tumor into one nonfragmented piece, and complete resection (R0 resection), defined as complete tumor removal with negative margins established, and curative resection, defined as an R0 resection with submucosal invasion less than 1000 *μ*m without lymphovascular involvement. The main outcome measures also included short-term and long-term complications. Short-term complications included bleeding, defined as hemorrhage accompanied by a decrease in hemoglobin of >2 g/dl from the baseline level or requiring an endoscopic hemostasis or transfusion, and perforation, defined as extraintestinal tissue projecting through a hole during treatment and/or the presence of parenteral gas as free or retroperitoneal air on postoperative abdominal radiographs. Long-term complications included local recurrence, defined as detection of dysplastic or neoplastic tissue at the scar. Metachronous tumor was defined as dysplastic/neoplastic lesion detected in another colon site during follow-up. Location of lesions was divided into the right colon (including cecum, ascending colon and transverse colon) and left colon (including descending colon, sigmoid colon and rectum). According to the Paris classification, polypoid lesions including pedunculated and sessile lesions and nonpolypoid lesions including superficial elevated, flat and depressed lesions. In our meta-analysis, laterally spreading tumor was classified to the nonpolypoid lesion.

### 2.4. Quality Assessment

The methodological quality of the included studies was assessed by Downs-Black quality checklist, which was designed to ensure the quality of both randomized and nonrandomized studies [10]. The checklist provides an overall numeric score of 30 points based on 5 domains as follows: reporting (overall quality), external validity (ability to generalize findings), bias (intervention and outcome measures), confounding (bias in sampling), and power (negative findings). Two reviewers independently evaluated the quality results of each study independently, and a final score for each study was resolved by discussion.

### 2.5. Statistical Analysis

We followed the methods of Xiu-He et al. [11]. All data analyses in this study were performed with software Stata, version 15.0. For the continuous outcomes including size of lesions and procedural time, its mean and variance were estimated from the median, range, and size of a sample [12], and the standard difference (SD) was calculated. The prevalence of the outcome variables in each study was combined to yield a pooled prevalence with a 95% confidence index (CI). A random-effects model was applied to generate a more conservative estimate of the prevalence. Cochran's *Q* test and an inconsistency index (*I*^2^) were used to assess the heterogeneity among studies. Heterogeneity was present if the *p* < 0.05 for Cochran's *Q* test, and *I*^2^ tests were used to assess the degree of heterogeneity (*I*^2^ < 25%, no heterogeneity; *I*^2^ = 25% − 50%, low heterogeneity; *I*^2^ = 50% − 75%, moderate heterogeneity; *I*^2^ > 75%, high heterogeneity) [13].

## 3. Results

### 3.1. Study Selection

**Figure 1:**
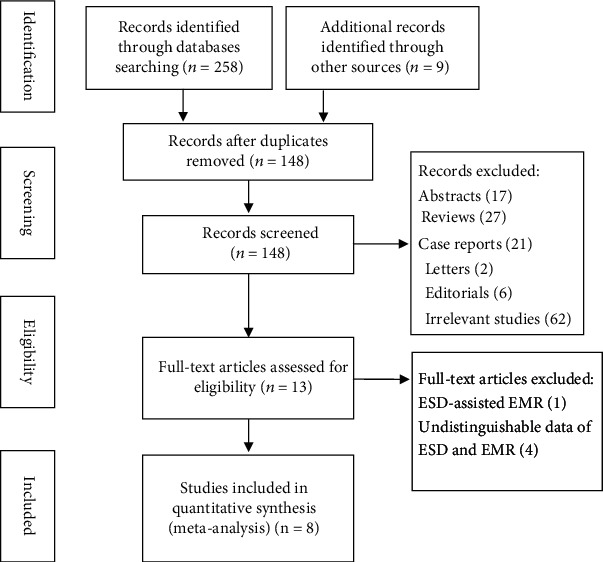
Flow diagram of the studies included in this meta-analysis.

A flow diagram of the systematic review is shown in Figure 1. After an initial search, 267 studies were identified. Then, 148 studies were screened after duplicates were removed. Of these articles, 13 studies were selected for further full-text evaluation after a review of the titles and abstracts. Of the 13 records, 8 studies fulfilled the criteria for inclusion in a quantitative synthesis (meta-analysis) [14–21].

### 3.2. Characteristics of Included Studies and Lesions

The included studies were published between 2015 and 2021. Among these studies, 4 studies were retrospective single-center case-control trials, 2 studies were retrospective multicenter cohort trials, and 2 studies were prospective multicenter cohort trials. There were a total of 192 UC patients and 203 dysplastic lesions. More male patients were discovered in the included studies. The total M/F ratio was 114/78. The median age was 61 years. The median duration of UC was 17 years. About 73% (112/154) of the UC patients were extensive colitis. The mean lesion size was 26.7 mm. Only 7 studies reported the location, morphology, surrounding mucosa, and submucosal fibrosis of lesions. About 83% (125/150) of the lesions were located in the left-side colon, 90% (135/150) of the lesions were nonpolypoid, 98% (198/203) surrounding mucosas of the lesions were in clinical/endoscopic remission, and about 71% (117/164) of the lesions had submucosal fibrosis. Only 4 and 7 studies reported the surface and border of lesions, respectively. All lesions from the reported studies were nonulcerative and well-defined. All included studies were assessed as moderate to high quality according to the criteria from the Downs-Black quality checklist. The study characteristics of the included studies are shown in Table 1. The clinical and technical characteristics are presented in Table 2.

### 3.3. Meta-Analysis Results

**Figure 2:**
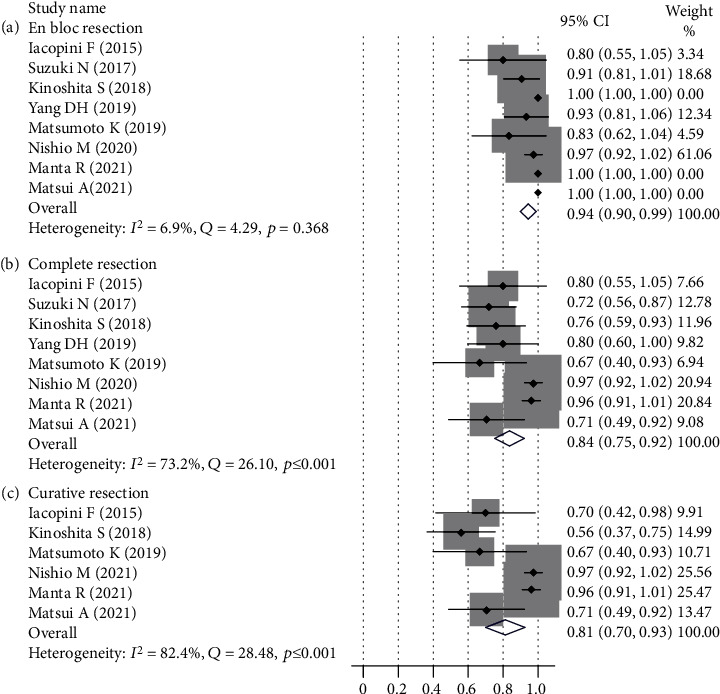
Resection rates and pooled estimates of endoscopic submucosal dissection.

An en bloc resection rate was reported in all 8 studies, and the pooled prevalence was 94% (95% CI (90%-99%)), which is shown in Figure 2(a), with no heterogeneity detected among the studies (*I*^2^ = 6.9%, *Q* = 4.29, *p* = 0.368). A complete resection (R0 resection) rate was also reported in 8 studies. The pooled prevalence was 84% (95% CI (75%-92%)), which is shown in Figure 2(b). A moderate heterogeneity was detected in the analysis of the complete resection rate (*I*^2^ = 73.2%, *Q* = 26.10, *p* ≤ 0.001). The curative resection rate was only reported in 6 studies. The pooled prevalence was 81% (95% CI (70%-93%)), which is shown in Figure 2(c). A high heterogeneity was detected in the analysis (*I*^2^ = 82.4%, *Q* = 28.48, *p* ≤ 0.001). The procedural time of ESD was only reported in 7 studies. The mean procedural time of ESD was 83 minutes.

**Figure 3:**
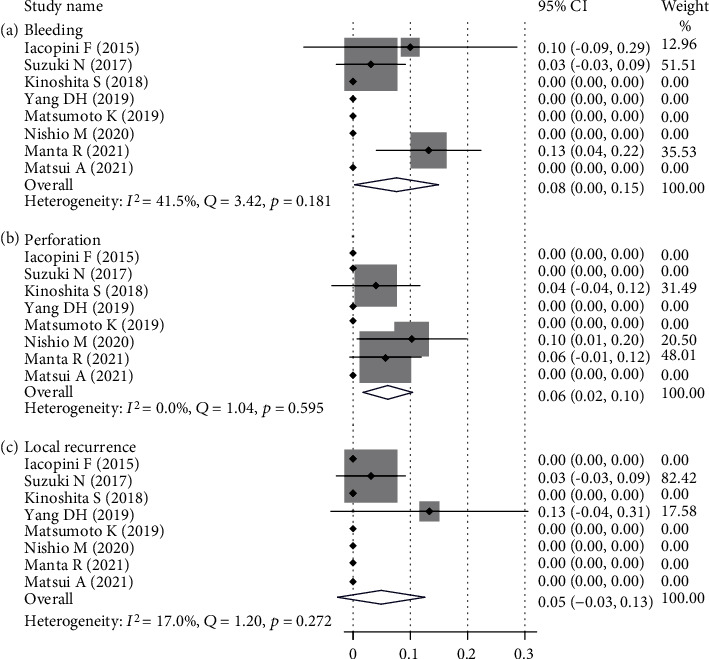
Prevalence of complications and pooled estimates of endoscopic submucosal dissection.

**Figure 4:**
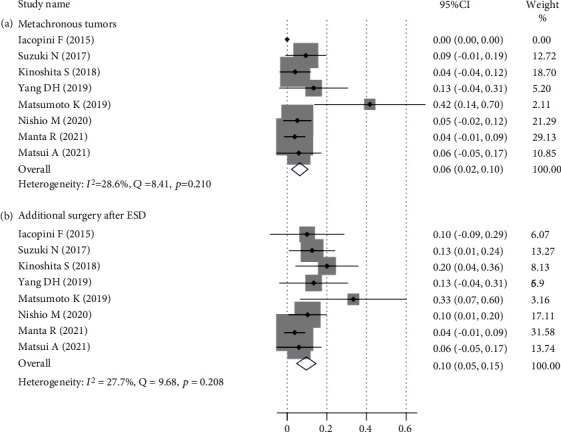
Prevalence of metachronous tumors and additional surgery after endoscopic submucosal dissection.

As shown in Table 3, bleeding and perforation were the main short-term complications in ESD of dysplasia in UC patients. The pooled prevalence of bleeding was 8% (95% CI (0%-15%)), which is shown in Figure 3(a), with a low heterogeneity (*I*^2^ = 41.5%, *Q* = 3.42, *p* = 0.181). The pooled prevalence of perforation was 6% (95% CI (2%-10%)), which is shown in Figure 3(b). No heterogeneity was detected (*I*^2^ = 0.0%, *Q* = 1.04, *p* = 0.958). During the follow-up period, the pooled prevalence of local recurrence was 6% (95% CI ((-3%)-13%)), which was shown in Figure 3(c), with no heterogeneity (*I*^2^ = 17.0%, *Q* = 1.20, *p* = 0.272). The pooled prevalence of metachronous tumors was 6% (95% CI (2%-10%)), which is shown in Figure 4(a), with a low heterogeneity (*I*^2^ = 28.6%, *Q* = 8.41, *p* = 0.21). The pooled prevalence of additional surgery after ESD was 10% (95% CI (5%-15%)), which is shown in Figure 4(b), with a low heterogeneity (*I*^2^ = 27.7%, *Q* = 9.68, *p* = 0.208).

## 4. Discussion

UC patients carry a higher risk of developing CRC through the inflammation–dysplasia–carcinoma sequence [5, 20]. Therefore, there is a chance to reduce the risk of CRC by identifying and treating dysplasia. ESD is a safe, effective, and well-established resection technique for superficial colorectal tumor [7]. This procedure allows en bloc resection for precancerous lesions and early cancers regardless of the lesion size, leading to a minimized recurrence risk [22]. However, submucosal fibrosis due to chronic inflammation in UC patients may increase the procedural risk and reduce the complete resection rate. So far, only fragmentary information from a few case series was about the outcomes of ESD for dysplasia in UC patients. Therefore, a pooled data analysis would be necessary and useful.

The colorectal submucosa in UC patients often present diffuse fibrosis (about 71% in our meta-analysis), which makes it difficult to obtain adequate mucosal lifting by submucosal injection and difficult to recognize the safe submucosal depth for dissection. Therefore, ESD for dysplasia in UC patients is full of technically challenge. However, our meta-analysis revealed that the en bloc resection rate, complete resection rate, and curative resection rate were 94%, 84%, and 81%, respectively. These results are comparable with the en bloc resection rate (89%-92%), complete resection rate (76%-83%), and curative resection rate (67.2%-84.1%) in ESD for sporadic CRC [23–25]. Meanwhile, the mean procedural time of ESD in UC patients (83 min) was not longer than that of ESD in sporadic CRC (75-106 min) [24, 25]. As for the safety of ESD for dysplasia in UC patients, our meta-analysis revealed that the incidences of bleeding, perforation, and local recurrence were 8%, 6%, and 5%, respectively. They were slightly higher compared with those for sporadic CRC (bleeding 2.7%, perforation 5.2%, and local recurrence 2%) [23]. Fortunately, all complications were successfully resolved by endoscopic/conservative treatment. Therefore, despite the existence of submucosal fibrosis, the procedure-related outcomes of ESD for dysplasia in UC patients are comparable with those of ESD for sporadic CRC, especially the resection rates. This may be related to the technique of dissection. According to Mizuno et al. [26], most dysplasia has a thin, clear layer at the bottom of the submucosa. Dissection at the bottom of the submucosa is the key to ensure a safe procedure. In addition, appropriate conditions for ESD for dysplasia in UC are also crucial [27]. First of all, the perilesional mucosa should be in remission endoscopically or the patient should be at least in clinical remission. Second, lesions should be with distinct border and no surface ulceration. Third, any of the endoscopic findings indicating possible invasive cancer should be absent, although the diagnostic performance of invasive pit or vascular patterns and the nonlifting sign has not been determined yet. Last but not the least, endoscopists should be highly skilled in colorectal ESD.

UC is a long-lasting and relapsing inflammatory bowel disease that can involve the entire colon and even the distal ileum. And, the development of CRC in UC follows the inflammation-dysplasia-carcinoma sequence [5, 20], which is different from sporadic CRC. Thus, in addition to the site of ESD, any part of the colon that is currently, or was previously, inflamed is at risk for neoplastic transformation [28, 29]. As our meta-analysis showed that the rate of metachronous tumors was 6%, therefore, ESD, in spite of its high complete resection rate, is not sufficient for dysplasia in ulcerative colitis due to the metachronous recurrence. Based on the pathogenesis of dysplasia, medications that target the inflammation are crucial to prevent the occurrence of dysplasia. Meanwhile, our meta-analysis also revealed that the rate of additional surgery after ESD were 10%. The rate of additional surgery after ESD was much higher compared with that for sporadic CRC (0.4%-1.1%) [23, 24]. Furthermore, the reasons for additional surgery after ESD were metachronous tumors (57%, 13/23), noncurative resection (39%, 9/23), and failed ESD (4%, 1/23). Among the metachronous tumors, 13 cases were treated by colectomy, and 3 cases were treated by ESD. So, ESD of dysplasia in UC patients is not a one-time deal; colectomy or another ESD may be needed.

The clinical outcomes of ESD for colorectal dysplasia in UC patients were fully evaluated through our meta-analysis. However, on the one hand, varying degrees of heterogeneities existed in the outcomes, especially the R0 resection rate and curative resection rate, which showed significant heterogeneity (*I*^2^ > 50%). The definitions of R0 resection and curative resection were clear and consistent among the included studies. However, factors that affected the resection rates were different among the studies. In our meta-analysis, the reasons for R0 resection and noncurative resection were severe submucosal fibrosis [14, 16, 18, 19], deep submucosal invasion of cancer [14–16], and positive horizontal margin [15, 21]. The different reasons for non R0 resection among these studies might be the source of heterogeneity. As for the complications of ESD for colorectal dysplasia in UC patients, a low heterogeneity was showed for the rate of bleeding, while no heterogeneity were found for rates of perforation and local recurrence. The reason for heterogeneity of bleeding rate might be the inconsistent definitions of bleeding among the included studies. For example, in Iacopini's study [14], bleeding was defined when endoscopic treatment was needed. While in Suzuki's study [15], the endoscopic treatment was not required. On the other hand, in order to compare the outcomes of ESD for colorectal lesions in UC patients and non-UC patients, indirect comparison with the data from published reports was conducted. Because of no controlled studies, a certain risk of bias is inevitable. Therefore, although the outcomes of ESD for dysplasia in UC patients were quantitatively combined and compared with sporadic CRC, these results should be interpreted with caution. In the end, the median follow-up duration was 29 months, which was relatively short for accurate estimates of recurrence and CRC incidences, future studies with a longer follow-up time are in need to verify the pooled estimates.

## 5. Conclusion

Results from this systematic review suggest that ESD is a safe and effective treatment for dysplasia in UC patients. Current literature data support the safety and effectiveness of this procedure for treatment of dysplasia in UC patients with comparable resection rates and acceptable complications incidences, compared to non-UC patients. Unfortunately, all the evidences are from observational studies; therefore, in the future, randomized controlled multicenter studies with less heterogeneity and longer follow-up are needed to better assess the clinical outcomes of ESD in UC patients.

## Figures and Tables

**Table 1:** Study and population characteristics of the included studies.

Author	Year	Country	Patients (*n*)	Lesions (*n*)	Median age (year)	Gender (M/F)	Median duration (year)	Extent of colitis (*n*, E/L/P)	Type of study	Center of study	Quality score
Iacopini F et al. [14]	2015	Italy, Japan	9	10	62	4/5	13	6/3/0	Prospective, cohort	Multicenter	19
Suzuki N et al. [15]	2017	United Kingdom, Japan	32	32	65	18/14	20	NA	Retrospective, cohort	Multicenter	23
Kinoshita S et al. [16]	2018	Japan	25	25	62	18/7	19	19/3/3	Retrospective, cohort	Multicenter	20
Yang DH et al. [17]	2019	South Korea	15	15	60	10/5	14	13/2/0	Retrospective, case control	Single-center	25
Matsumoto K et al. [18]	2019	Japan	7	12	55	5/2	15	4/2/1	Retrospective, case control	Single-center	19
Nishio M et al. [19]	2020	Japan	39	39	56	22/17	17	30/0/9	Retrospective, case control	Single-center	24
Manta R et al. [20]	2021	Italy	53	53	65	31/22	17	30/23/0	Prospective, cohort	Multicenter	22
Matsui A et al. [21]	2021	Japan	12	17	59	6/6	20	10/2/0	Retrospective, case control	Single-center	20

M/F: male/female ratio; UC: ulcerative colitis; E/L/P: extensive colitis/left-side colitis/proctitis; NA: not available.

**Table 2:** Clinical and technical characteristics of the included studies.

Author	Mean size (mm)	Location (*n*, R/L)	Morphology (*n*, P/NP)	Surface ulcer (*n*)	Border (*n*, clear/unclear)	Surrounding mucosa (*n*, R/A)	Submucosal fibrosis (*n*, Y/N)	Mean procedure type (min)	En bloc resection (*n*)	Complete resection (*n*)	Curative resection (*n*)	Histopathology (*n*)
Iacopini et al. [14]	36.25	2/8	0/10	0	10/0	10/0	9/1	75.25	8	8	7	SSA 1, LGD 4, HGD 3, adenocarcinoma 2
Suzuki et al. [15]	33	0/32	2/30	NA	32/0	29/3	31/1	87	29	23	NA	LGD 19, HGD 7, adenocarcinoma 4, regenerative atypia 2
Kinoshita et al. [16]	21.6	8/17	5/20	NA	25/0	25/0	25/0	71.7	25	19	14	LGD 7, HGD 4, adenocarcinoma 14
Yang et al. [17]	26.5	1/14	1/14	0	15/0	15/0	10/5	73.5	14	12	NA	SSA/P 1, IND 1, LGD 8, HGD 3, adenocarcinoma 2
Matsumoto et al. [18]	18.25	0/12	2/10	NA	12/0	10/2	12/0	52.5	10	8	8	LGD 9, HGD 3
Nishio et al. [19]	19	12/27	4/35	0	39/0	39/0	NA	67	38	38	38	LGD 17, HGD 13, serrated polyps 9
Manta R et al. [20]	34	NA	NA	0	NA	53/0	29/24	NA	53	51	51	LGD 37, HGD 14, IND 1 hyperplastic polyp 1
Matsui et al. [21]	25.1	2/15	1/16	NA	17/0	NA	1/16	155	17	12	12	Adenoma 2, LGD 4, HGD 4, adenocarcinoma 7

NA: not available; R/L: right colon/left colon; P/NP: polypoid/nonpolypoid; R/A: remission/active; Y/N: yes/no; LGD: low-grade dysplasia; HGD: high-grade dysplasia; IND: indefinite dysplasia; SSA/P: sessile serrated adenoma/polyp. Right colon including cecum, ascending colon, and transverse colon. Left colon including descending colon, sigmoid colon, and rectum. Polypoid including pedunculated and sessile lesions. Nonpolypoid including superficial elevated, flat and depressed lesions, and laterally spreading tumor.

**Table 3:** Complications and follow-up results in the collected studies.

Author	Complications (*n*)	Median follow-up (month)	Local recurrence (*n*)	Metachronous tumors (*n*)	Additional surgery after ESD (*n*)
Iacopini et al. [14]	Bleeding 1	24	0	0	1
Suzuki et al. [15]	Bleeding 1	33	1	3	4
Kinoshita et al. [16]	Perforation 1	21	0	1	5
Yang DH et al. [17]	No	25	2	2	2
Matsumoto K et al. [18]	No	180	0	5	4
Nishio M et al. [19]	Perforation 4	37	0	2	4
Manta R et al. [20]	Bleeding 7, perforation 3	37	0	2	2
Matsui A et al. [21]	No	25	0	1	1

ESD: endoscopic submucosal dissection.
